# Differential activation of AKT isoforms by growth factors in human myotubes

**DOI:** 10.14814/phy2.15805

**Published:** 2023-10-25

**Authors:** Brandon M. Roberts, Alyssa V. Geddis, Ronald W. Matheny

**Affiliations:** ^1^ US Army Research of Environmental Medicine Natick Massachusetts USA; ^2^ Military Operational Medicine Research Program Ft. Detrick Maryland USA

**Keywords:** anabolic, myosin, PKB, protein synthesis, skeletal muscle

## Abstract

AKT signaling plays a crucial role in muscle physiology, and is activated by stimuli, including insulin, growth factors, and exercise. Three AKT isoforms have been identified in mammals, and they possess both distinct and redundant functions. However, it is currently unknown what the predominant AKT isoform is in primary human skeletal myotubes, and very little is known regarding the effects of insulin and insulin‐like growth factor‐I (IGF‐I) on AKT isoforms activation in human myotubes. Thus, we sought to determine the abundances of each AKT isoform in primary human skeletal myotubes and their responses to insulin or IGF‐I. Analysis of protein lysates by liquid chromatography‐parallel reaction monitoring/mass spectrometry revealed that AKT1 was the most abundant AKT isoform and AKT3 was the least‐abundant isoform. Next, myotubes were treated with either 100 nM insulin or 10 nM IGF‐I for 5, 20, 45, or 60 min. In response to insulin, there was a significant treatment effect on phosphorylation of AKT1 and AKT2, but not AKT3 (*p* < 0.01). In response to IGF‐I, there was a significant treatment effect on phosphorylation of pan‐AKT at all timepoints compared to control (*p* < 0.01). Next, we determined how much of the total AKT isoform pool was phosphorylated. For insulin stimulation, AKT1 was significantly higher than AKT2 at 5 min and 60 min posttreatment (*p* < 0.05 both) and significantly different than AKT3 at all timepoints (*p* < 0.05). For IGF‐I stimulation, AKT1 was significantly higher than AKT2 at 45 and 60 min posttreatment (*p* < 0.05 both) and significantly higher than AKT3 at all timepoints (*p* < 0.05). Our findings reveal the differential phosphorylation patterns among the AKT isoforms and suggest a potential explanation for the regulatory role of AKT1 in skeletal muscle.

## 1 INTRODUCTION

AKT is a serine/threonine kinase, also known as protein kinase B (PKB), which mediates cellular processes including proliferation, metabolism, and differentiation in skeletal muscle. There are three AKT family members—AKT1, AKT2, and AKT3—which are structurally similar and are activated by common biochemical pathways. In human skeletal muscle, AKT1 and AKT2 are the main isoforms expressed, and it was previously shown that AKT2 is the predominant AKT isoform expressed in humans (Matheny et al., 2018). However, it is currently unknown what the main isoform is in primary human skeletal myotubes or if these AKT isoforms are differentially regulated by growth factors such as insulin‐like growth factor‐I (IGF‐I) or insulin.

In animal experiments, the role of the three AKT isoforms has been identified using knockout mice. AKT1 is expressed in all tissues and appears to regulate growth as AKT1 knockout mice have smaller body size without any other noticeable phenotype (Cho, Mu, et al., 2001; Cho, Thorvaldsen, et al., 2001). AKT2 knockout mice have impaired glucose tolerance and reduced insulin‐stimulated glucose transport, but AKT1 and AKT3 knockout mice do not, suggesting AKT2 controls glucose homeostasis. Furthermore, dual systemic knockout of AKT1 and AKT2 in mice result in severe muscle hypoplasia incompatible with postnatal life (Peng et al., 2003; Yang et al., 2005). AKT3 is primarily expressed in the brain, and AKT3 knockout mice have reduced brain size with no other phenotype (Easton et al., 2005). Taken together, in mice, it appears that AKT1 controls growth, AKT2 controls glucose metabolism, and AKT3 plays a role in the brain; however, there may be overlapping AKT functions between AKT1 and AKT2 isoforms (Dummler et al., 2006; Easton et al., 2005).

AKT isoforms have also been studied in vitro. In C2C12 cells, AKT1 and AKT2 contribute to myoblast differentiation, although AKT1 has been shown to play a more prominent role than AKT2 (Gardner et al., 2012). In C2.7 myoblasts, AKT1 is essential for cell proliferation, while AKT2 promotes cell cycle exit (Héron‐Milhavet et al., 2006). In C3H10T1/2 mouse embryonic fibroblasts, AKT1 is necessary for initiation and maintenance of myoblast differentiation but AKT2 is dispensable (Wilson & Rotwein, 2007). These studies have examined AKT isoforms in various cell lines, but little information is available about their abundance or how they are activated by IGF‐I or insulin, thus making it difficult to translate findings from cell culture models to humans.

Previous studies have manipulated AKT signaling in primary human myoblasts and myotubes without fully understanding their response to stimulus or the amount of each AKT isoform (Matheny et al., 2018). Therefore, the purpose of this experiment was to determine the most abundant AKT isoform expressed in cultured primary human skeletal myotubes. Then, determine how insulin or IGF‐I stimulation affects phosphorylation of the three AKT isoforms along with the fraction of total AKT phosphorylated. We hypothesized that like humans, AKT2 would be the predominant isoform in primary human myotubes. We further hypothesize that AKT2 will be phosphorylated to a larger degree than AKT1 or AKT3 when stimulated with IGF‐I or insulin.

## 2 METHODS

### 2.1 Materials and reagents

Human Skeletal Myoblasts (#A11440 or #A12555), low glucose Dulbecco's Modified Eagle Medium (#11885092), Horse Serum (#26050070), Bovine Serum Albumin (#A9576), and Pierce A/G Magnetic Beads (#88802) were purchased from Thermofisher Scientific Antibodies for Total AKT1 (#2938), Total AKT2 (#2964), Total AKT3 (#14293), Total AKT (#4685), p‐AKT S473 (#4060), p‐AKT1 S473 (#9018), p‐AKT2 S474 (#8599), AKT (Pan) (C67E7) (rabbit monoclonal; #4691), AKT (Pan) (40D4) (mouse monoclonal; #2920), and Human Insulin‐like Growth Factor 1 (#8917) were purchased from Cell Signaling Technologies. Secondary antibodies Anti‐Rabbit IgG HRP‐Linked Antibody (PN#7074) and Anti‐Mouse IgG HRP‐Linked Antibody (PN#7076) were purchased from Cell Signaling Technologies. Human Insulin Solution (#I9278) was purchased from Sigma Aldrich ImageJ was downloaded from the National Institutes of Health website.

### 2.2 Cell culture

Cells for experiments were from two different donors: one Caucasian male (#A11440) and one Caucasian female (#A12555). Each experiment was completed using both cell donors in technical triplicate. Human skeletal myoblasts were differentiated at 37°C and 5% CO_2_ as previously described (Matheny et al., 2022). Cells were tested for mycoplasm, and the results were negative. Human IGF‐I was diluted in 20 mM citrate and human insulin solution (insulin) was diluted in phosphate‐buffered saline (PBS). Cells were exposed to 100 nM insulin (0.005%) or 10 nM IGF‐I (0.1%). Cells were differentiated in Dulbecco's modified Eagle medium (DMEM) supplemented with 2% horse serum for 72 h. Cells were serum starved in DMEM supplemented with bovine serum albumin (BSA) for 120 min, followed by exposure to 10 nM IGF‐I or 100 nM Insulin for 5 , 20 , 45 , or 60 min.

### 2.3 Protein extraction, immunoprecipitation, and immunoblotting

Cellular protein extraction, Bradford analysis and immune blotting, was conducted as previously described (Matheny et al., 2015; Roberts et al., 2023). Immunoprecipitation was performed to identify phosphorylated and total AKT3. Whole cell lysate (250 ug) was immunoprecipitated using protein A/G magnetic beads and an antibody specific for phosphorylated AKT S473 or total AKT3. Immune blotting was conducted using the immunoprecipitated sample and an antibody for phosphorylated AKT S473 or total AKT3. Densiometric quantification analysis was done using NIH ImageJ 1.60 (Kolb et al., 2021).

To determine the role of each AKT isoform in the IGF‐I and insulin response, primary human myotubes were treated with either 100 nM insulin or 10 nM IGF‐I for 5, 20, 45, or 60 min and analyzed for AKT phosphorylation by western immunoblotting. AKT phosphorylation was determined using an AKT antibody that detects phosphorylation of all AKT isoforms in the hydrophobic motif (HM) (Serine 473 in AKT1, Serine 474 in AKT2, and Serine 472 in AKT3). Isoform‐specific AKT1 and AKT2 phosphorylation was determined using antibodies specific for AKT1 S473 or AKT2 S474, while phosphorylation of AKT3 was determined by immunoprecipitating lysates using an AKT3‐specific antibody followed by western blotting using the AKT HM antibody.

### 2.4 Liquid chromatography‐parallel reaction monitoring/mass spectrometry

Liquid chromatography‐parallel reaction monitoring/mass spectrometry (LC‐PRM/MS) was performed as previously described with a rabbit and mouse pan AKT antibody (Matheny et al., 2018). Briefly, samples were eluted from the beads from the immunoprecipitation by heating in LDS Sample Buffer (ThermoFisher Scientific). The mobility region was excised and processed by in‐gel digestion with trypsin. Excised gel bands were first washed with ammonium bicarbonate followed by acetonitrile, then reduced followed by alkylation, digested with trypsin, and quenched with formic acid. Peptides were lyophilized and reconstituted. Half of each digested sample was analyzed by LC‐PRM/MS.

### 2.5 Statistics

**FIGURE 1:**
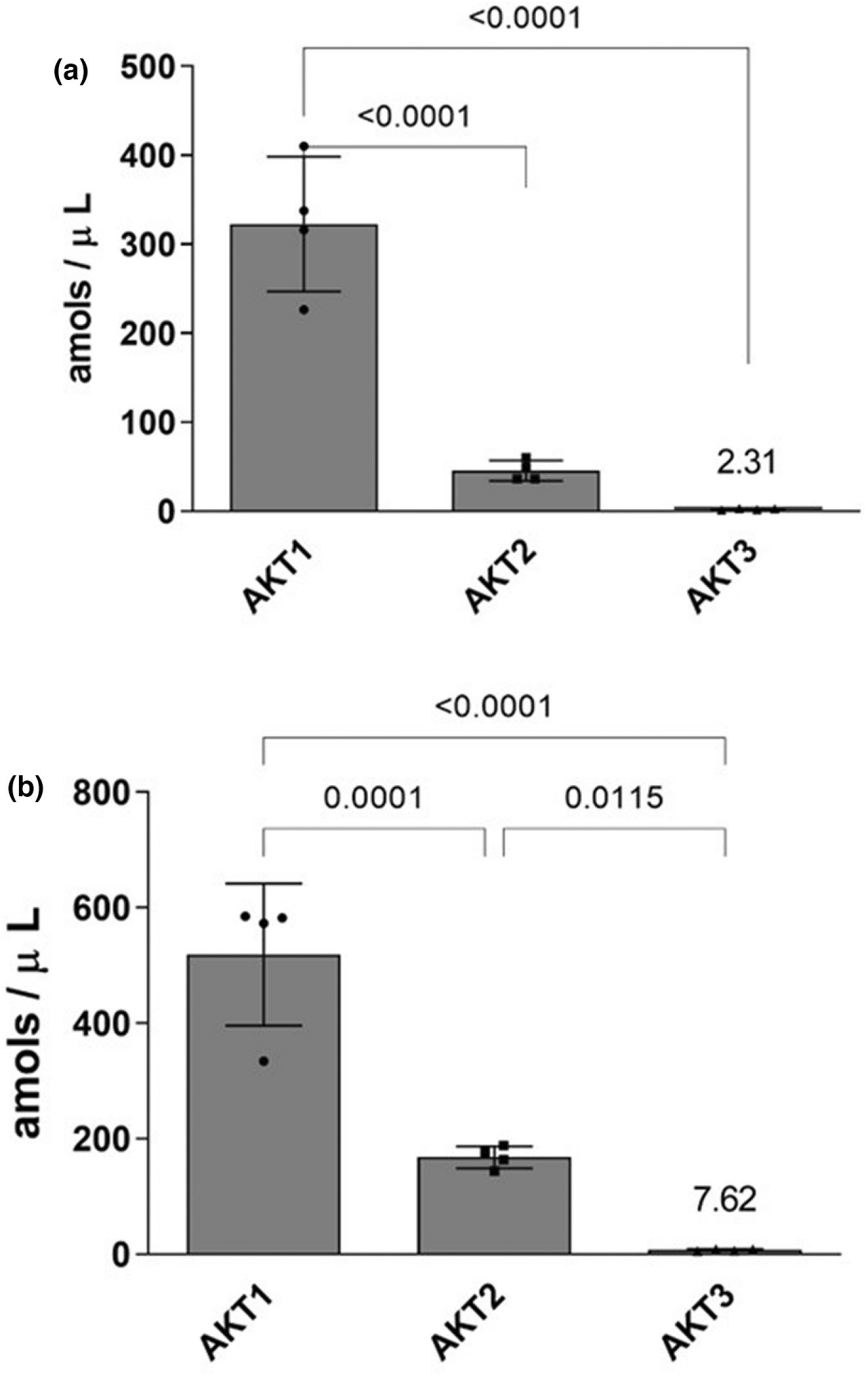
AKT abundance primary human skeletal myotubes as measured by immunoprecipitation followed by liquid chromatography‐parallel reaction monitoring/mass spectrometry (LC‐PRM/MS). Immunoprecipitation was performed on lysates derived from cultured human myotubes using either (a) rabbit or (b) mouse pan AKT antibodies followed by analysis by LC‐PRM/MS. Data represented as individual points and Mean ± SD.

**FIGURE 2:**
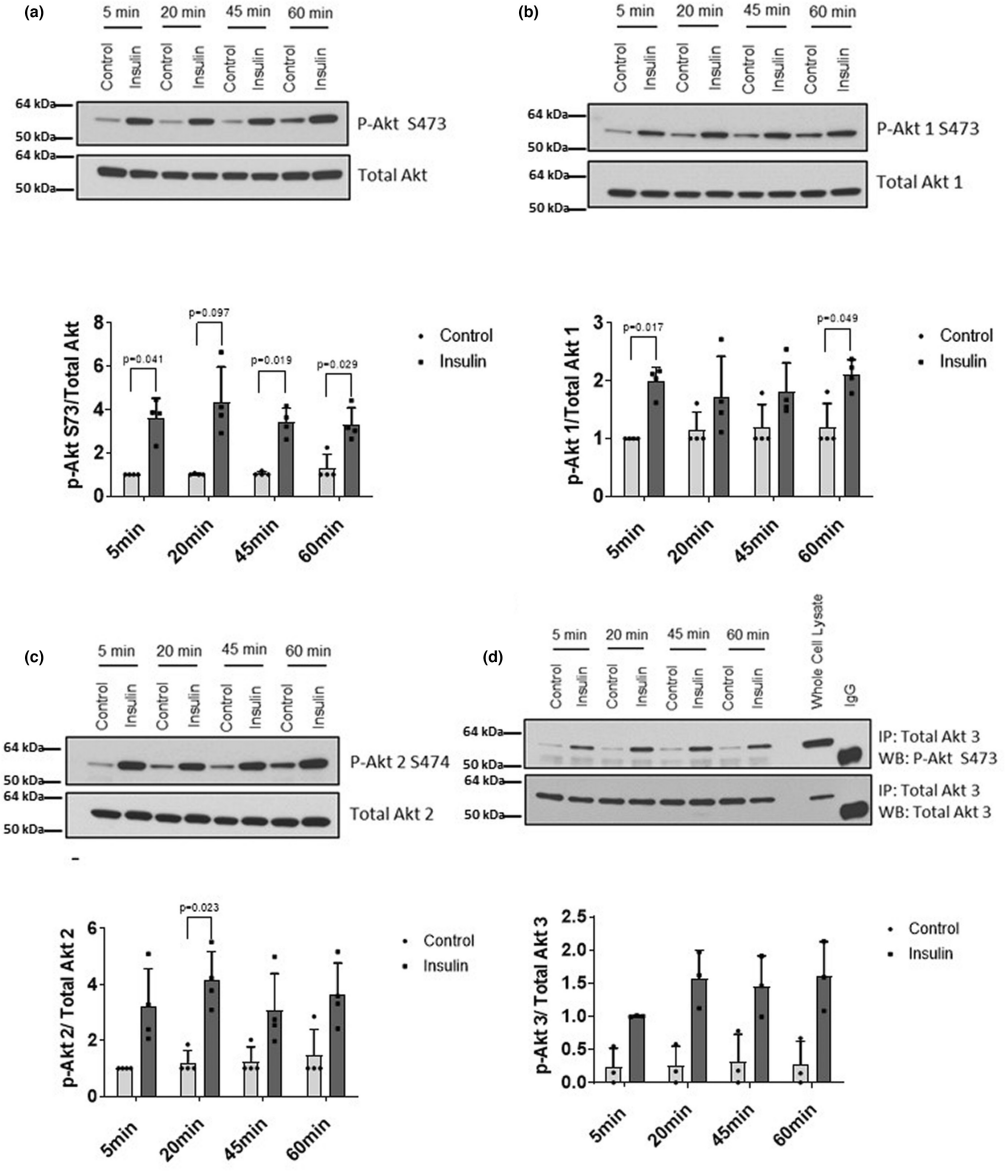
Effects of insulin on phosphorylation of AKT isoforms in cultured human myotubes. Myotubes were serum‐starved for 120 min and then treated with 100 nM insulin (or vehicle) for the indicated times (*n* = 3). Western immunoblotting was performed using (a) p‐AKT, (b) p‐AKT1, (c) p‐AKT2 and (d) p‐AKT3. Whole cell lysate (250 μg) was immunoprecipitated with AKT3‐specific antibody followed by western blot using either phospho‐Akt S473 or total AKT3 as primary antibodies to detect phospho‐AKT3 or total AKT3, respectively. Whole cell lysate (25 μg) as well as 15 μg normal rabbit IgG were simultaneously subjected to western blotting to verify band identities. Data represented as individual data points and Mean ± SD.

**FIGURE 3:**
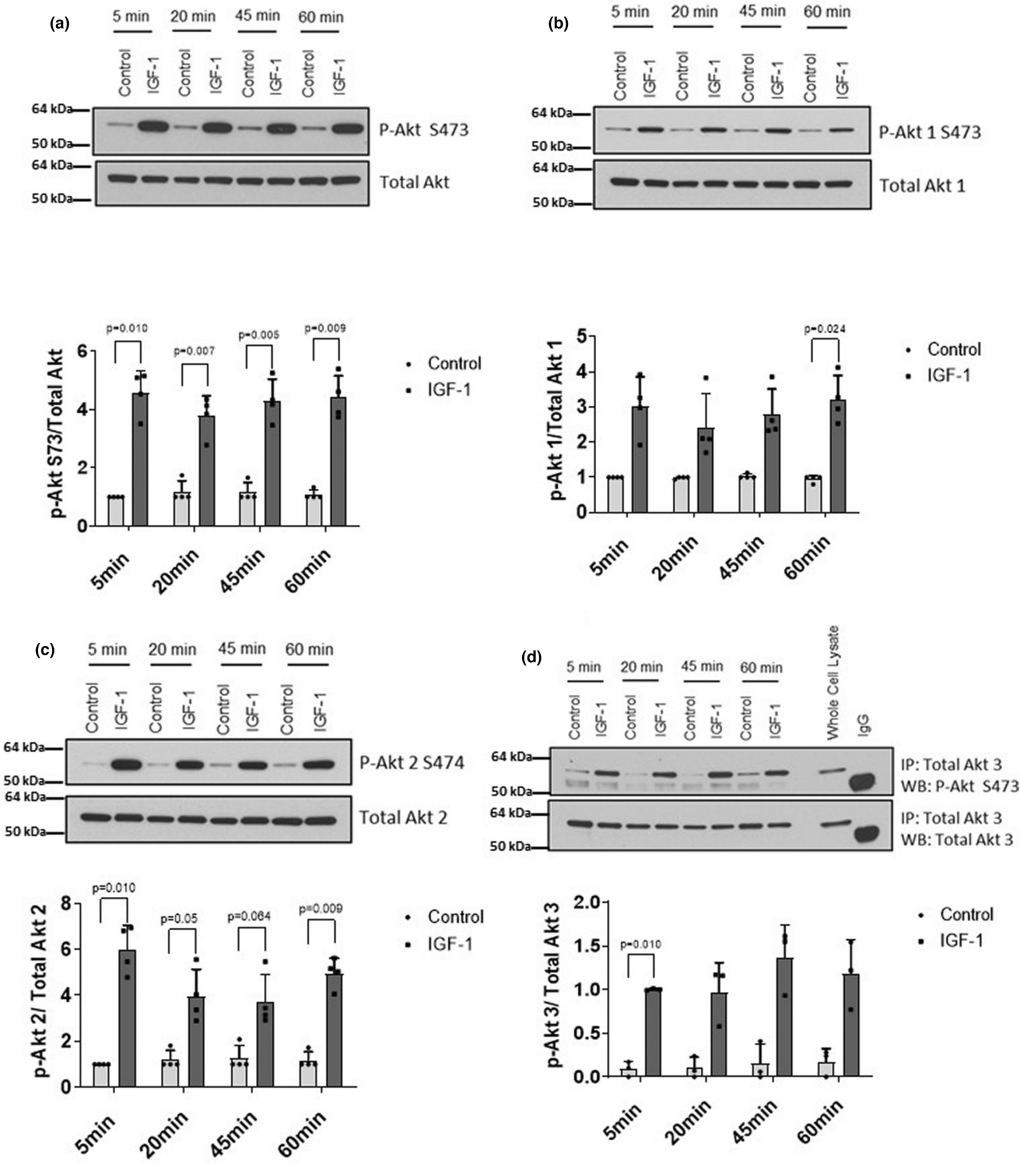
Effects of insulin‐like growth factor‐I (IGF‐I) on phosphorylation of AKT isoforms in cultured human myotubes. Myotubes were serum‐starved for 120 min and then treated with 100 nM IGF‐I (or vehicle) for the indicated times. Western immunoblotting was performed using (a) p‐AKT, (b) p‐AKT1, (c) p‐AKT2, and (d) for p‐AKT3. Whole cell lysate (250 μg) was immunoprecipitated with AKT3‐specific antibody followed by western blot using either phospho‐Akt S473 or total AKT3 as primary antibodies to detect phospho‐AKT3 or total AKT3, respectively. Whole cell lysate (25 μg) as well as 15 μg normal rabbit IgG were simultaneously subjected to western blotting to verify band identities. Data represented as individual data points and Mean ± SD.

Data are presented as mean ± SEM. Statistics were performed using one‐way ANOVA with Dunnett's multiple comparison test performed post hoc for the AKT concentrations (Figure 1). A two‐way ANOVA was completed for phosphorylated AKT isoforms stimulated with IGF‐I or Insulin (Figures 2 and 3) with post hoc Fisher's LSD test. *p* < 0.05 was considered significant. Experiments were completed in triplicate (*n* = 3).

## 3 RESULTS

Analysis of protein lysates with rabbit pan AKT revealed that AKT1 (518.75 amols/uL) was the most abundant AKT isoform, expressed ~3‐fold greater than AKT2 (168.22 amols/uL, *p* < 0.001). AKT3 was the least‐abundant AKT isoform, expressed at a level ~ 2% that of AKT1 (7.62 amols/uL *p* < 0.001) (Figure 1a). Analysis of protein lysates with mouse pan AKT indicated AKT1 (322.28 amols/uL) was the most abundant AKT isoform, expressed ~7‐fold greater than AKT2 (45.59 amols/uL, *p* < 0.001). AKT3 was the least abundant AKT isoform, expressed at a level ~ 1% that of AKT1 (2.31 amols/uL, *p* < 0.001) (Figure 1b).

In response to insulin, there was a significant treatment effect on phosphorylation of AKT at the HM with a 2.5‐ to 4.3‐fold increase at all timepoints compared to vehicle‐treated control myotubes (Figure 2a, *p* < 0.05). There was also a significant effect of insulin treatment on phosphorylation of AKT1, which was elevated 1.5‐ to 2‐fold at all timepoints (Figure 2b, *p* < 0.05), and AKT2 (2.4‐ to 3.4‐fold greater than control (Figure 2c, *p* < 0.05)). Insulin‐stimulated AKT3 phosphorylation was not significantly different than control at any timepoint (Figure 2d).

In response to IGF‐I, there was a significant treatment effect on phosphorylation of AKT at the HM demonstrating a 4‐ to 5‐fold increase at all timepoints compared to controls (Figure 3a, all timepoints *p* < 0.01)). There was a significant treatment effect of IGF‐I on phosphorylation of AKT1, which was elevated 2.5‐ to 3.4‐fold at 5, 45, and 60 min posttreatment (Figure 3b, *p* < 0.05). Phosphorylation of AKT2 was 3‐ to 6‐fold greater than control and significant at all timepoints (Figure 3c, *p* < 0.05). AKT3 phosphorylation 5‐ to 10‐fold greater than control, but not significant at any timepoint (Figure 3d).

**FIGURE 4:**
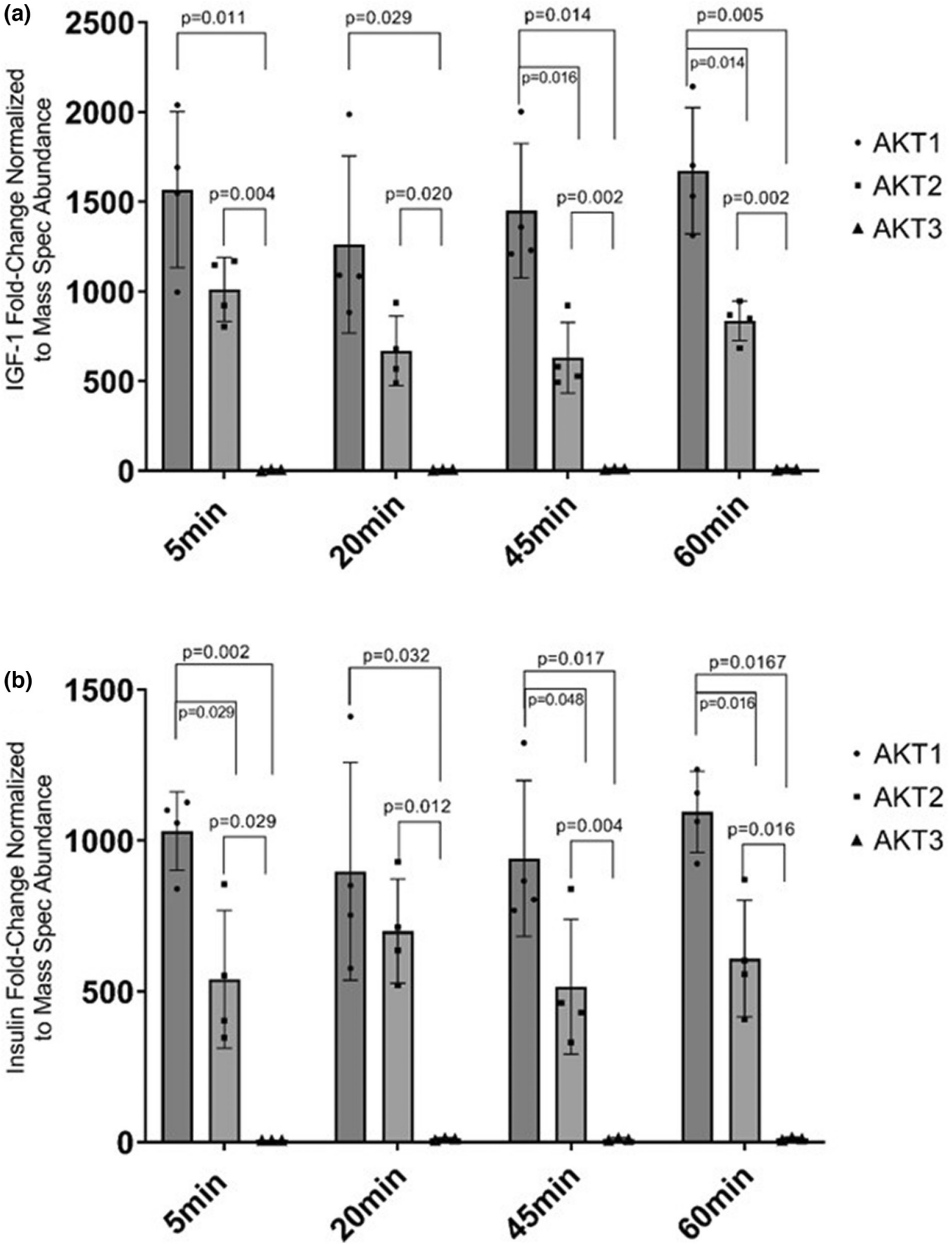
Phosphorylated AKT fold‐changes normalized to total protein abundance. Myotubes were serum‐starved for 120 min and then treated with (a) 100 nM insulin or (b) 10 nM IGF‐I or vehicle for the indicated times. Fold‐changes were quantified for phosphorylated/total AKT then normalized to the total amount of AKT found in the LC‐PRM/MS measurement. Data represented as individual data points and Mean ± SD.

Given the LC‐PRM/MS and phosphorylation results, we next determined how much of the total AKT isoform pool was phosphorylated. To accomplish this, we divided the phosphorylated fold‐change data from IGF‐I or insulin stimulation by the total AKT pool from the LC‐PRM/MS results from the rabbit antibody. For insulin stimulation, AKT1 was significantly higher than AKT2 at 5 min and 60 min posttreatment (Figure 4a, *p* < 0.05 both) and significantly different than AKT3 at all timepoints (Figure 4a, P < 0.05). AKT2 was significantly higher than AKT3 at all timepoints (Figure 4a, *p* < 0.05). For IGF‐I stimulation, AKT1 was significantly higher than AKT2 at 45 and 60 min poststimulation (Figure 4b, *p* < 0.05 both) and significantly higher than AKT3 at all timepoints (Figure 4b, *p* < 0.05). AKT2 was significantly higher than AKT3 at all timepoints (Figure 4b, *p* < 0.05).

## 4 DISCUSSION

In this study, we provide quantitative data regarding the abundance of AKT isoforms in primary human skeletal myotubes. Using LC‐PRM/MS, we determined that AKT1 was in greater abundance than AKT2, while AKT3 was virtually undetectable. We also found that AKT2 was phosphorylated more than AKT1 or AKT3 with IGF‐I or insulin when normalized to total protein via immunoblot. Upon adjusting for total protein levels using LC‐PRM/MS, our study revealed that the total pool of AKT1 exhibited higher phosphorylation compared to AKT2 and AKT3. In contrast to our hypothesis, these results indicate that AKT1 demonstrated a greater extent of phosphorylation compared to AKT2 and AKT3. This finding highlights the differential phosphorylation patterns among the AKT isoforms and suggests human primary tubes are differentially regulated compared to human skeletal muscle (Matheny et al., 2018).

A major function of AKT is to control insulin signaling (Jaiswal et al., 2019). AKT2 is thought to be essential for normal glycemia and the most important AKT isoform for insulin‐stimulated glucose transport (Cho, Mu, et al., 2001; Whiteman et al., 2002). For example, silencing of AKT2 gene expression using siRNA in human primary muscle cells abolishes the effects of insulin on glucose metabolism, emphasizing the role of AKT2 in regulating glucose metabolism specifically in human skeletal muscle (Bouzakri et al., 2006). A recent manuscript challenged the common belief that reduced AKT activity in skeletal muscle leads to systemic glucose intolerance and insulin resistance. It demonstrated that mice lacking AKT2 alone in skeletal muscle exhibited normal insulin signaling, glucose tolerance, and insulin sensitivity (Jaiswal et al., 2019). However, deletion of both AKT isoforms resulted in muscle atrophy and impaired downstream signaling, suggesting that AKT1 and AKT2 may have overlapping functions in response to insulin signaling (Jaiswal et al., 2019). Our results support this view as insulin treatment increased phosphorylation of both AKT1 and AKT2, which indicate potential redundancy in signaling. These findings suggest that alterations in additional signaling molecules, in addition to skeletal muscle AKT phosphorylation, may impact glucose tolerance and insulin sensitivity.

Insulin‐like growth factors, such as IGF‐I, are capable of promoting muscle differentiation in cell culture, and their actions through the IGF‐I receptor have been linked to the formation, regeneration, and growth of skeletal muscle in vivo (Florini et al., 1996; Matheny & Adamo, 2009; Matheny et al., 2010; Matheny & Nindl, 2011). Previous research indicates IGF‐I treatment of myoblasts cells leads to an increase in phosphorylation of AKT and to a rapid and sustained induction of AKT enzymatic activity (Tureckova et al., 2001). Other studies agree, demonstrating persistently enhanced phosphorylation of AKT in L8 and C2C12 myoblasts treated with IGF‐I (Li et al., 2000). Our data extend these findings, by identifying how each AKT isoform responds to IGF‐I treatment in primary human myotubes. We found phosphorylation of each AKT isoform from IGF‐I and insulin with the main difference being the magnitude of phosphorylation. Specifically, AKT1 demonstrated higher fold‐changes compared to AKT2 and substantially higher fold‐changes than AKT3. Furthermore, once adjusted for the total protein pool, the total pool of AKT1 was phosphorylated more than AKT2. This suggests that AKT1 phosphorylation is a key driver of the IGF‐I response in primary human skeletal myotubes.

Our experiments have strengths and limitations. First, we did not measure the Threonine 308 phosphorylation site of any AKT isoform. However, as full activation of AKT requires both T308 and S473, and localization of AKT to membrane‐bound PIP3 facilitates T308 phosphorylation by PDK1, and S473 phosphorylation by mTORC2, we consider phosphorylation of AKT at S473 a reasonable proxy for full AKT activation (Alessi et al., 1996). Thus, future research should identify the role of T308 in response to IGF‐I and insulin. Another limitation is that we only completed experiments in one cell type; therefore, future research should compare AKT mechanisms in various cell types so that we can develop better in vitro models prior to animal or human research. Finally, we did not attempt to determine if AKT1 and AKT2 regulation are redundant or compensatory in response to IGF‐I or insulin because it was beyond the scope of the current study. It should also be noted that the antibody used to immunoprecipitated AKT isoforms was raised against a sequence within the C‐terminus, and that the C‐termini of AKT isoforms do differ somewhat, depending on the region (Rehan et al., 2014). It should be kept in mind, however, that two distinct antibodies were used, and each resulted in nominally similar results (the abundance of AKT1 > AKT2 > AKT3). A strength of our manuscript is the specificity of our experiments to each AKT isoform, and the use of two different stimuluses (IGF‐I and insulin).

In summary, we found that among the three AKT isoforms in primary human skeletal myotubes, AKT1 was the most highly‐expressed AKT isoform, followed by AKT2, and AKT3 was the least‐abundant isoform. Insulin and IGF‐I phosphorylated AKT2 to a larger extent than AKT1, and this held true when normalized to via immunoblot, but not total protein pool via LC‐PRM/MS. Ultimately, defining the actions of specific AKT isoforms in human skeletal muscle is an important step in identifying AKT‐mediated processes that contribute to musculoskeletal health.

## CONFLICT OF INTEREST STATEMENT

The authors declare no conflicts of interest.
